# Depth-Related Visuomotor Performance in Keratoconus and Its Relationship to Stereopsis

**DOI:** 10.1167/iovs.66.4.31

**Published:** 2025-04-14

**Authors:** Preetirupa Devi, Christa M. Bhengra, Deepak Kumar, Rashmi Deshmukh, Pravin K. Vaddavalli, Joshua A. Solomon, Christopher W. Tyler, Shrikant R. Bharadwaj

**Affiliations:** 1 Centre for Applied Vision Research, City St. George's, University of London, Northampton Square, London, United Kingdom; 2 Brien Holden Institute of Optometry and Vision Sciences, L V Prasad Eye Institute, Hyderabad, Telangana, India; 3 Professor Brien Holden Eye Research Centre, Hyderabad Eye Research Foundation, L V Prasad Eye Institute, Hyderabad, Telangana, India; 4 Shantilal Sanghvi Cornea Institute, L V Prasad Eye Institute, Hyderabad, Telangana, India

**Keywords:** blur, contrast, phase disruption, retinal disparity, visuomotor, wavefront aberrations

## Abstract

**Purpose:**

The purposes of this study were to quantify the impact of degraded binocularity in keratoconus and its improvement with rigid contact lenses on a depth-related visuomotor task that emulates complex activities in daily living; and to determine whether visuomotor performance may be predicted from psychophysical estimates of stereo threshold.

**Methods:**

Participants were instructed to pass a metal loop around a wire convoluted in depth. Error rate and speed were measured in 26 controls, 30 cases with keratoconus with best-corrected spectacles, a subset of 17 cases with rigid contact lenses, and 10 uncorrected myopes with acuity and stereo thresholds comparable to the keratoconic cohort. Stereo thresholds were determined using random-dot stimuli.

**Results:**

Binocular error rates were lower than monocular error rates for controls, uncorrected myopes, and the better-performing half of cases (*p* < 0.001, for each), but not for the worst-performing half (*p* = 0.07). Error rates in cases improved with contact lenses (*p* < 0.001). Within each cohort, the error rate was poorly correlated with the stereo threshold (*r*^2^ < 0.12, for each). Monocular speeds were significantly lower than binocular speeds for controls than for cases (*p* = 0.003) and for uncorrected myopes than cases (*p* = 0.001).

**Conclusions:**

Degraded binocularity in keratoconus may limit the ability to perform depth-related visuomotor tasks. A portion of this loss may be overcome by using rigid contact lenses. The attributes of visuomotor task performance are, however, not predictable from the psychophysical estimates of stereo thresholds.

Consider the acts of inserting a key into a keyhole, placing a light bulb in its socket, or threading a needle. These seemingly straightforward activities of daily living are complex visuomotor tasks that require precise estimation of the spatial configurations for the planning and execution of appropriate hand movements and grasp actions.^1^^–^^3^ The visual system's ability to estimate 3D information, particularly for motor actions as opposed to perception,^3^ is largely governed by the processing of retinal disparity arising from the triangulation of both eyes onto the object of interest.^4^ The loss of binocularity arising from temporary occlusion or from the permanent loss of vision in one eye significantly impairs visuomotor performance.^1^^,^^5^ Similar results are observed with the deterioration of binocularity from optical blurring,^6^ pathologies like amblyopia,^7^^,^^8^ and macular degeneration.^9^ In general, task accuracy worsens and the speed of task performance decreases with degraded/absent depth vision, relative to viewing with intact binocularity.

This background led us to investigate the status of visuomotor task performance in the optical condition of keratoconus. This progressive ophthalmic disease, typically affecting individuals in their second to third decades of life,^10^ is characterized by spatial and depth vision losses arising from degraded retinal image quality caused by an abnormally shaped cornea of one or both eyes.^11^ The keratoconic eye's optical quality, when described using the Zernike polynomial series, shows elevated levels of coma, trefoil, and spherical aberrations.^12^^,^^13^ The resultant radially asymmetric blur produces significant contrast demodulation and “doubling” or “ghosting” of local image features due to optical phase shifts.^14^^,^^15^ Usually, even in bilateral keratoconus, the grade of disease and the topography is different between the two eyes, resulting in dissimilar blur patterns.^16^ The combination of the radial and bilateral asymmetry in blur significantly impacts the formation of the cyclopean image needed for processing binocularity.^14^^,^^15^ All grades of binocularity appear to be degraded in keratoconus, relative to age-similar controls: retinal disparity processing is impaired due to correspondence mismatches in the aberrated retinal images^14^; the worse of the two eyes may be suppressed,^17^ and stereo thresholds may be three-to-seven-fold worse, independent of keratoconus severity.^18^ Motor fusion and ocular accommodation may also be impaired in keratoconus, thereby preventing clear and single binocular vision at near viewing distances.^19^

Three specific objectives surrounding the impact of the optical limitations on the depth-related visuomotor task performance in keratoconus were investigated in the present study. The primary objective was to compare the monocular and binocular visuomotor task performance in keratoconic participants and similarly aged controls on a stereoscopic buzz-wire task. This task involves passing a metal loop around a wire that is convoluted in depth, avoiding contact as much as possible.^1^^,^^5^ Task performance is quantified in terms of the error rate (i.e. the frequency of contacts made between the loop and the wire per second, each of which is signaled by an audio-buzz) and the speed of loop movement along the wire. This task has been shown to reveal a greater difference between binocular and monocular viewing in controls than tasks like the peg board and bead threading because it limits the use of tactile feedback.^7^^,^^8^ We hypothesized that the degraded/absent binocularity in keratoconus would result in the error rate and speed of task performance becoming similar under monocular and binocular viewing conditions. The losses in spatial and depth vision arising from the degraded retinal image quality in keratoconus are typically managed using rigid contact lenses that replace the distorted cornea with a smoother refracting surface.^20^ Therefore, the second study objective tested the hypothesis that an improvement in retinal image quality using rigid contact lenses would result in a commensurate improvement in the buzz-wire task performance in keratoconus.

Although the status of binocularity may be investigated using several psychophysical paradigms, stereo thresholds obtained using dichoptic stereograms remain the most widely used measure in the clinic and in research investigations.^21^ Interestingly, the depth-related visuomotor task performance of individuals with amblyopia, strabismus, and in those with purposely induced degradations in binocularity have all revealed a negative correlation with their stereo threshold.^6^^–^^8^ Given this, the third study objective tested the hypothesis that binocular advantages would be smaller with high stereo thresholds in keratoconus.

## Methods

### Participants

Thirty participants with keratoconus (henceforth called “cases”) and 26 similarly aged participants without keratoconus (henceforth called “controls”) were recruited from the patient base and staff/student pool of the L. V. Prasad Eye Institute (LVPEI), Hyderabad, India. An a priori power analysis was conducted using G*Power version 3.1.9.4 for sample size estimation,^22^ based on data from Gonzalez et al.,^23^ which compared depth precision in 9 uniocular children with depth precision in 13 binocular children. The effect size in that study was 1.1, considered to be large using conventional criteria.^24^ With a significance criterion of α = 0.05 and power = 0.80, the minimum sample sizes needed with this effect size is *N* = 24 for a *t*-test between cases and controls, supporting the adequacy of our sample size of 30 cases and 26 controls.

The study adhered to the tenets of the Declaration of Helsinki and was approved by the Institutional Review Board of LVPEI. All participants signed a written informed consent form before study induction. Diagnosis of keratoconus was based on a comprehensive eye examination that showed evidence of keratoconus with objective, non-cycloplegic refraction, slit-lamp examination, and corneal tomography. Standard clinical management was followed for all cases, with no influence of the study protocol on their clinical care. If necessary, keratoconus was managed with rigid contact lenses as per standard operating protocols.^25^ Disease severity was determined using the D-index, a multimetric measure of the corneal structural deformation, obtained using Scheimpflug imaging tomography (Pentacam HR, Oculus Optikgeräte; Wetzlar, Germany).^26^ The D-index was derived for both eyes of all participants using the Belin-Ambrósio enhanced ectasia display map and included deviations of front and back surface elevations of the cornea, pachymetric progression, thinnest corneal point, and deviation of Ambrósio relational thickness maximum.^26^ This metric has been shown to have good reliability in the diagnosis and progression of keratoconus, with higher D-index values indicating greater disease severity.^27^

The best spectacle-corrected, high contrast, monocular distance visual acuity in each eye, as estimated using the routine clinical protocol, ranged from 0.00 to 1.60 logMAR in cases. The equivalent acuity values were all 0.00 logMAR in controls (20/20; visual acuity beyond 0.00 logMAR is typically not measured in the clinical protocol at the institute where the study is conducted). All cases and controls had monocular near acuities between 0.00 and 0.40 logMAR (N8) at 40 cm. Unaided visual acuity was not recorded in this study. Participants with any other ophthalmic dysfunction, or any systemic condition that resulted in restricted body movement, visible shaking of hands, inability to follow instructions, or inability to fuse the stereogram for stereopsis measurements, were excluded.

Seventeen cases were habitual rigid contact lens users [one case wore a Rose K2 lens (Menicon Co. Ltd., Nagoya, Japan), while the rest wore conventional rigid gas permeable lenses (Purecon McAsfeer, Silver line laboratory Pvt. Ltd, India)] (see Appendix). Based on the severity and requirement for contact lenses, 11 participants wore contact lenses in both eyes and the rest wore these lenses only in one eye (see Appendix). The lenses were fitted by experienced contact lens practitioners at LVPEI, using the manufacturers’ recommended protocols, and the final lenses were ordered and dispensed to the participants as a part of regular clinical protocol. The visual acuity, stereo thresholds, and the buzz-wire performance were tested both before and after contact lens fitting. The visual acuities ranged from 0.00 to 0.40 logMAR with their contact correction.

### The Buzz-Wire Apparatus and Task Performance

**Figure 1.**
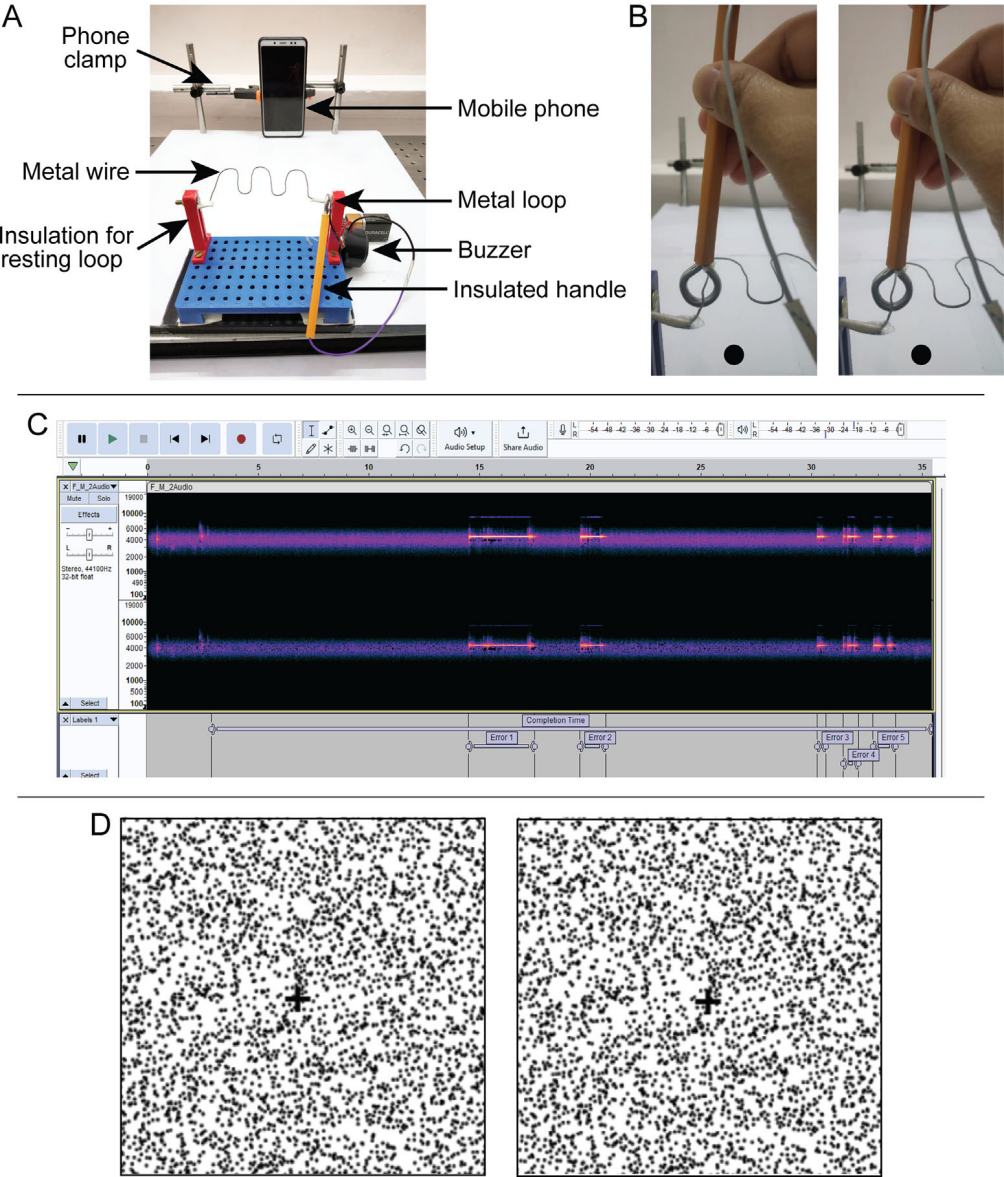
(**A**) The buzz-wire apparatus from the participant's viewpoint with the key elements highlighted. (**B**) A representative, stereoscopic photograph depicting the position of the metal loop around the wire track. Readers can cross-fuse the two images to view the pattern in 3D. (**C**) A representative spectrogram used for the audio analysis of the buzzes using the Audacity software. The spectrogram shows the labels marked for the completion time and for the epochs of error time stamps (high contrast tracks in the spectrogram) during a representative trial. (**D**) A representative, cross-fusible, example of the random-dot stereogram used for estimating the stereo threshold. The fused stereogram shows a leftward tilted rectangular bar in crossed retinal disparity.

The buzz-wire apparatus and task have been described in detail by Devi et al.^5^ Briefly, the apparatus was composed of a 33.5 cm long wire of 1 mm diameter shaped into three horizontal depth curves, with its edges clamped onto vertical posts (Fig. 1A). The wire pattern was mounted parallel to the horizontal plane, resulting in continuous changes in depth from one end to the other (free-fuse the stereo pair in Fig. 1B to experience the depth impression). A 10 mm diameter metal loop, held by hand with a 9 cm long stalk, was guided along the wire and delivered an auditory buzz each time the loop came in contact with the wire (Fig. 1A). Three buzz-wire apparatuses with similar amounts of depth modulation but different wire patterns due to slight phase shifts were employed in this study to assess task reproducibility. Devi et al.^5^ determined that if the wire were to be at the center of the loop in the buzz-wire task, the gap between the wire and one end of the loop would subtend a mean diastereopsis disparity of 611 arc sec (range = 450–715 arc sec, depending on the participant's interpupillary and viewing distances; see fig. 7 in Devi et al).^5^ The entire apparatus, the participants’ face, and the experimental surrounding were video recorded using the front camera of a standard cellular phone (Redmi Note 5 Pro, Xiaomi, China) that was fixed to a custom-built clamp at 30 cm from the buzz-wire apparatus (field of view captured by the phone camera = 42 degrees × 55 degrees).

Participants were positioned 30 cm away from the buzz-wire at an average elevation angle of −45 degrees (inter-participant range depending on their height = 36–53 degrees; Fig. 1A), so that it provided both monocular and stereoscopic cues to its convolutional structure. The buzz-wire task was described as a “game” to the participants, with the following instructions given at the beginning of the game, verbatim in English or in their local language:

“Look at the camera without moving for 5 seconds, during which I will give a verbal countdown and say START, upon which you will start the game. Your task is to pass the loop from one end to the other end without touching the wire. In case the loop touches the wire, you will hear the buzzer ring. When you hear the buzzer, stop your movement, and make the buzzing stop by centering the wire within the circular loop. Once the buzzing stops, proceed forward until you reach the other end. Make sure the loop is held upright throughout the game.”

No explicit instructions were provided to the participants on the speed with which they needed to play the game. The instructions were reiterated at the beginning of each experimental trial. The instructions were accompanied by the examiner demonstrating each step to ensure the participants understood what should and should not be done.^28^^,^^29^ However, no prior practice trials were given to the participants to retain the difference in the viewing conditions.^7^ The direction of movement of the loop, that is, from the left end to the right end of the wire or vice versa — was randomized at the beginning of each trial. All participants performed the buzz-wire task under binocular and monocular viewing conditions. They performed the task thrice for each viewing condition with different patterns of the wire formation, all in random order. For monocular viewing of controls, one eye was randomly occluded, while the worse eye (based on visual acuity) of cases was occluded to minimize the impact of resolution loss on task performance. In cases with equal acuity in both eyes, one eye was randomly occluded. Their heads remained free to move during the task. Each run took approximately 40 seconds to complete, following which participants were given 1-minute break prior to the next trial.

The trial began once it was ensured that the participant was looking straight at the camera in the apparatus (Fig. 1A). The task performance in each trial was recorded for offline analysis. After task completion, the examiner manually checked every video to discard trials where the participant dragged the loop along the wire, a strategy deemed invalid for task completion. The accepted video files were then analyzed using custom-written software in Python (version 3.10, Centrum voor Wiskunde en Informatica, Amsterdam, The Netherlands). The videos were first cropped from the beginning of the task to its end, as determined by the examiner's verbal utterance of the word START to the metal loop entering the insulated portion of the wire on the other end. The videos were then analyzed for buzzes using the open-source Audacity software (version 3.2.1, Audio.com, Boston, MA, USA; Fig. 1C). The spectrogram of the audio signal generated by the movement of the loop along the wire, including the buzzes, was then bandpass filtered to a frequency range of 4 to 4.1 kHz. Intensities outside this frequency range were cut off at −30 dB to differentiate buzzes from the background noise (Fig. 1C). The total number of buzzes and the time stamps corresponding to the onset and termination of each buzz were then computed for the entire video.

### Estimation of Outcome Variables From the Buzz-Wire Task

The elapsed time between the beginning and end of the video file was deemed as the total task duration (in seconds). Error rate was calculated as the frequency of occurrence of the error buzzes over the total task duration (in errors/second). The speed at which the task was completed, when the participant was not making an error, was calculated as the length of the wire (33.5 cm) divided by the error-free time (in cm/second). The error-free time, in turn, was calculated as the total task duration minus the total time spent in making the errors (each error epoch was defined as the elapsed time between the start and end of the error buzz). The binocular advantage in error rate was calculated as the ratio of the monocular to binocular error rate (in case of zero error rate, the respective values were arbitrarily replaced by 0.001, as described in Devi et al.^5^). Similarly, the binocular advantage in speed was calculated as the ratio of binocular to monocular speed. In both cases, a ratio greater than unity indicated superior performance under binocular than monocular viewing.

### Measurement of Stereo Threshold

Stereo threshold was measured at a 50 cm viewing distance using random-dot stimuli presented on a gamma calibrated LCD monitor (1680 × 1050 pixel resolution, 59 hertz [Hz] refresh rate) and controlled using the Psychtoolbox-3 interface of MATLAB (R2016a; The MathWorks, Natick, MA, USA).^30^ The random-dot stimuli incorporated a rectangular disparity-defined bar oriented either with a leftward or a rightward tilt in crossed retinal disparity (Fig. 1D). The dichoptic stimuli were fused using a handheld stereo viewer with built-in periscopic mirrors to adjust for the participant's horizontal phoria and interpupillary distance (Screen-Vu Stereoscope, Portland, OR, USA). Vertical phoria, if any, was corrected with minor adjustments in head orientation. Data collection began once the participant reported stable fusion of the bounding box that presented the random-dot stimuli (Fig. 1D). Participants identified the direction of the bar tilt for every stimulus presentation while the retinal disparity varied in a two-down and one-up adaptive staircase manner with each presentation. For a better visibility of the stereoscopic rectangular bar, the initial disparity value was set anywhere between 2000 and 4000 arc sec. Until the first reversal, the disparity was changed by 50% of the previous disparity value. At the subsequent reversals, the disparity changed with a 5% step size. The staircase was terminated after 11 reversals. Response frequencies were fit with Weibull functions to obtain maximum-likelihood estimates^31^ and credible intervals for the 70.7% correct threshold level.

### Protocol

The buzz-wire and stereo tasks were performed by all participants with natural pupils and accommodative states. Among the cases, the first measurements were always made with their habitual spherocylindrical spectacles and then with their habitual rigid contact lenses, if any. The measurements were made in this order so as to not to deform the cornea with the rigid contact lens wear, which, in turn, would alter the pattern of retinal image blur experienced by the participant.^32^ Change in the monocular task performance with contact lens wear was not determined in this study.

### Schematic Framework for Data Interpretation

**Figure 2.**
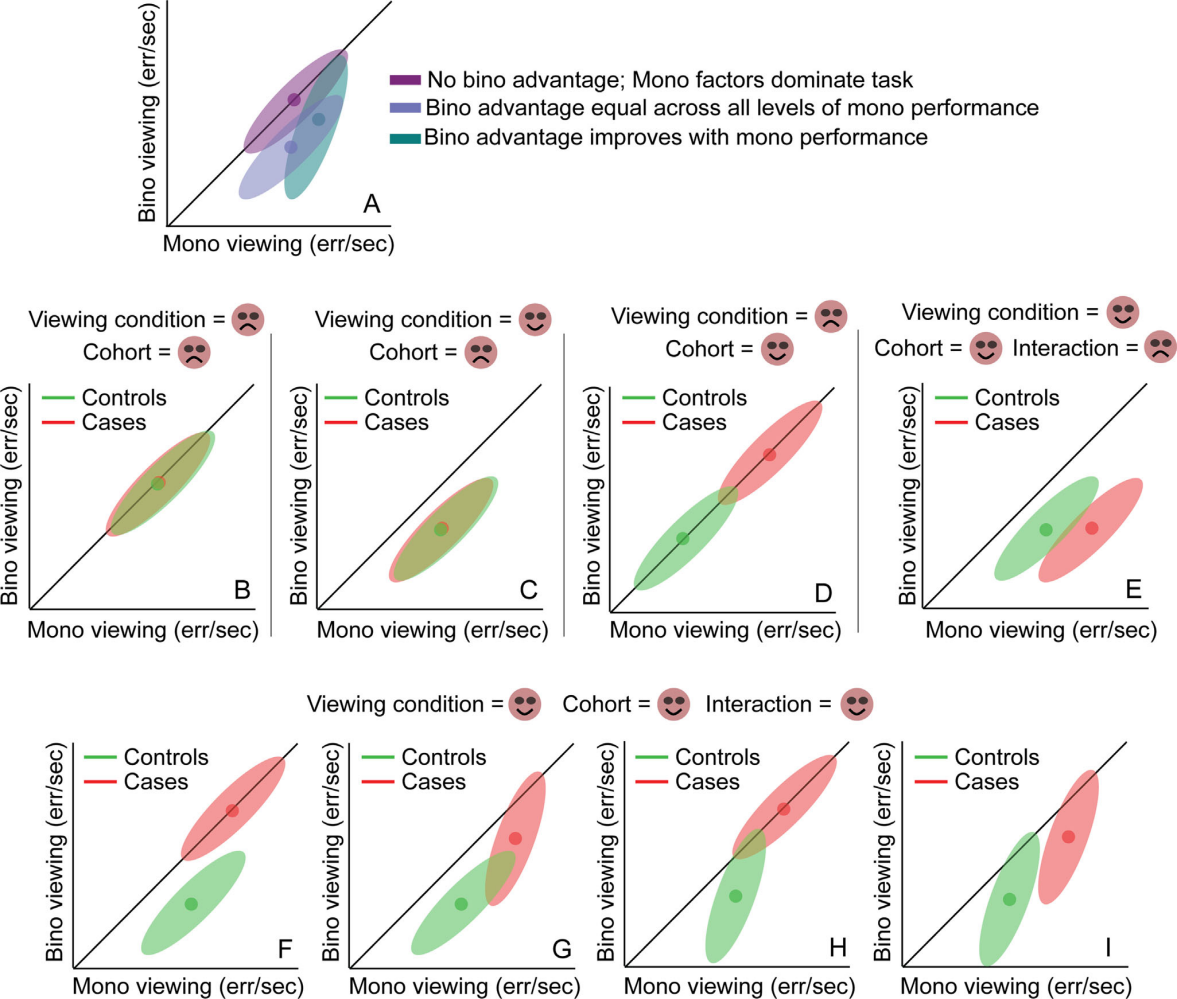
Schematics for the different pattern of results that may be obtained across controls and cases for the error rates in the buzz-wire task used in this study. Data clouds are assumed to have elliptical distributions. The *solid circle* is the centroid of the elliptical data cloud. The *“smiley” face* indicates statistically significant impact of the independent variable (i.e. viewing condition) on the dependent variable (i.e. error rate, in this case), whereas the *“gloomy” face* indicates no evidence of such a statistical significance. Panels **A** and **B** through **I** are described in the text.

To enable ease of interpretation, the data clouds obtained for error rate and speed in controls and cases were fit with bivariate contour ellipses using plot_ellipse.m code in Matlab.^33^ The x- and y-coordinates of the centroid and the major axes of the ellipses were determined from the fits. These outcomes were interpreted in the context of a schematic framework described below (Fig. 2A). In this schematic, the binocular and monocular error rates are plotted against each other. Whereas the 45 degrees line of equality indicates no binocular advantage and thus dominance of the task by monocular factors (purple cloud in Fig. 2A), data below this line would indicate a performance advantage derived from binocular depth cues (e.g. retinal disparity) and/or from the integration of monocular cues (e.g. occlusion and perspective cues) from the two eyes. The data could be uniformly distributed below the line of equality, indicating a uniform binocular advantage across the range of monocular error rates (blue cloud in Fig. 2A). The orientation of the data could also be steeper than 45 degrees, indicating that the binocular and monocular error rates are becoming more and more similar, with an increase in the monocular error rates (turquoise cloud in Fig. 2A). That is, the binocular advantage in error rate reduces with an increase in the monocular error. This could indicate that the binocular advantage in error rates may be determined by factors that limit the monocular performance in this task (e.g. retinal image quality, in this case) or simply that the error rates have reached the maximum that could be measured by the apparatus.

A range of possible comparisons between controls and cases is further illustrated in Figures 2B to 2I. The data of cases and controls may overlap along the line of equality, indicating no impact of viewing condition or cohort on task performance (Fig. 2B). The data clouds may remain overlapped but with both shifted below the line of equality, indicating a significant impact of only viewing condition but not cohort on task performance (Fig. 2C). The data clouds may also appear translated along the equality line, indicating a significant impact of cohort (cases producing more errors than controls in this schematic) but not of viewing condition on task performance (Fig. 2D). The data clouds may be shifted below the line of equality and appear horizontally translated relative to each other, indicating significant impact of both viewing condition and cohort but with no interaction between the factors (Fig. 2E). Figures 2F to 2I show data clouds wherein the main effect of both factors and the interaction between them are significant. In Figure 2F, the binocular advantage is present only for controls and not for cases. In Figures 2G and 2H, the binocular advantage is present for both cohorts, but only one cohort shows a monocular dependence of the binocular advantage – cases in Figure 2G and controls in Figure 2H. Finally, in Figure 2I, the binocular advantage in error rates show monocular dependence, but to varying extents, in both cohorts. These data schematics can also be extrapolated to the speed of task performance wherein faster movement under binocular viewing is indicated by the data lying above the line of equality (schematic not shown here).

### Data Analyses

**Figure 3.**
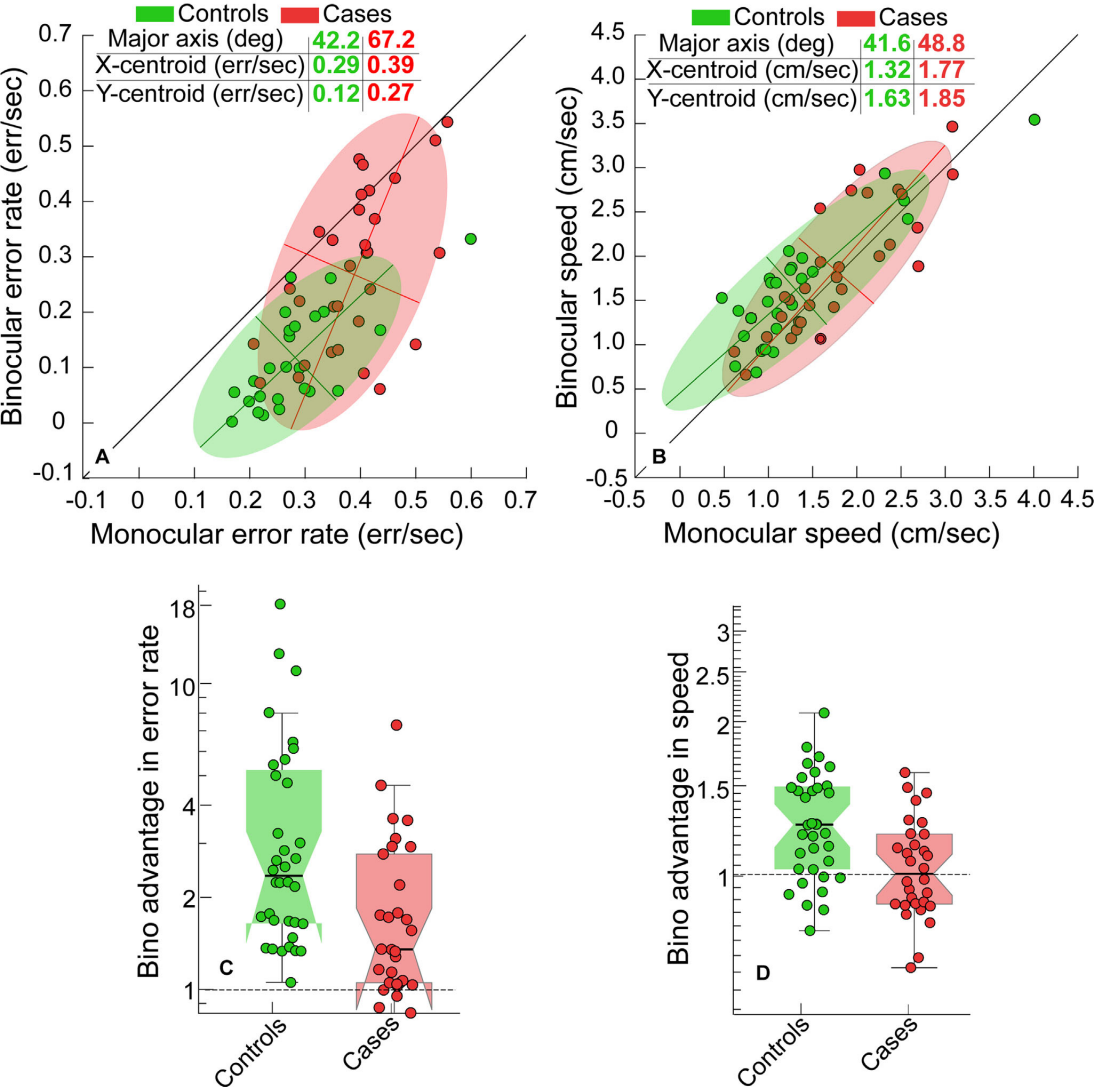
Scatter diagrams of the error rate (**A**) and speed (**B**) obtained from controls (*green symbols*) and cases (*red symbols*) while performing the buzz-wire task in this study. The colored patches represent the best-fit bivariate contour ellipse for the controls and cases datasets. The major and minor axes are shown for each ellipse, the intersection of which represents its centroid. The *diagonal line* in each panel represents the line of equality for monocular and binocular performance. The gestalt obtained from these contours may be readily compared with the schematics described in Figure 2. Panels **C** and **D** show the Box and Whisker plots of the binocular advantage in error rate and speed obtained for controls and cases in this study, respectively. For each box and whisker plot, the horizontal line is the median, the edges are the 25^th^ and 75^th^ quartiles and the whiskers are the 1^st^ and 99^th^ quartiles. The *green* and the *red dots* are the individual data points, jittered randomly along the X-axis for ease of visualization.

**Figure 4.**
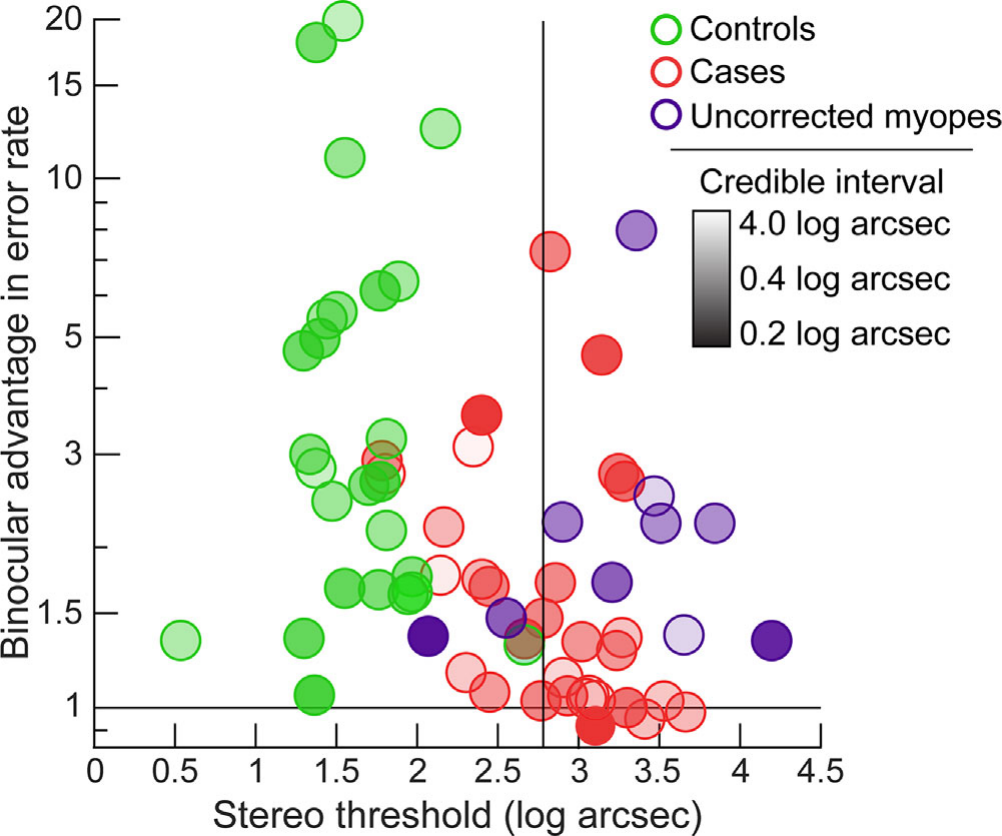
Binocular advantage in error rate plotted against the random-dot stereo threshold for controls (*green*), cases (*red*), and uncorrected myopes (*blue*). The transparency of the dots represents the 68% credible interval for the stereo thresholds. The *vertical line* indicates the disparity threshold (611 arc sec or 2.79 log arc sec) for diastereopsis.^5^^,^^23^ The *horizontal line* denotes the level where there was no binocular advantage.

**Figure 5.**
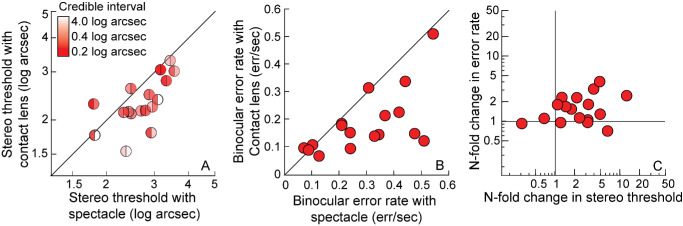
(**A,** **B**) Show the stereo threshold and error rate, respectively, obtained with the spectacle and contact lens corrections in cases. The transparency on the right and left hemispheres in **A**, represents the 68% credible interval for the spectacle and contact lens, respectively. (**C**) Shows the fold-change in stereo threshold from spectacles to contact lens wear plotted against the corresponding fold-change in error rates of the buzz-wire task. The region above the intersection of the *vertical* and *horizontal lines* indicates an improvement in both parameters with contact lens wear in this panel. The region diagonally opposite this indicates worsening of performance in both parameters with contact lens wear.

**Figure 6.**
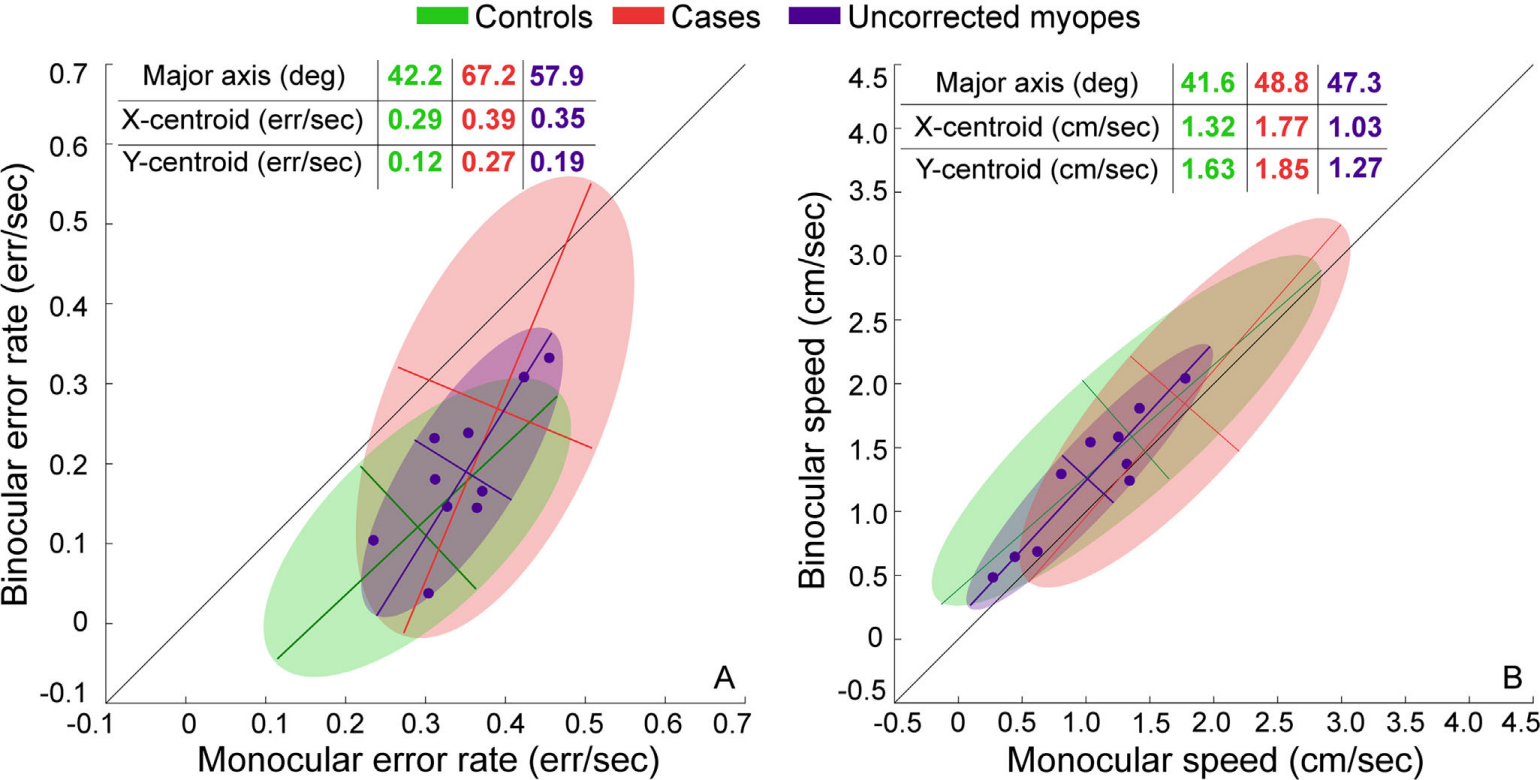
Scatter diagrams of the error rate (**A**) and speed (**B**) in uncorrected myopes (*blue symbols*) while performing the buzz-wire task plotted along the corresponding bivariate contour ellipses. The ellipses of the controls (*green*) and cases (*red*), identical to those in Figure 3, are also reproduced here for comparison purposes. All other details are the same as Figure 3.

Statistical analyses were performed using IBM SPSS Statistics (version 21; Armonk, NY, USA), Matlab (R2016a), and Wolfram Mathematica (version 14.1.0, Wolfram Research, Inc., Champaign, IL, USA). Because there were no overall trends in the error rate or speed across the three repetitions of the buzz-wire task,^5^ these quantities were averaged for further analyses. The Shapiro-Wilk test revealed that error rate, speed, and the binocular advantage of error rate and speed were non-normally distributed. Hence, the datasets of error rate and speed were Box-Cox transformed using a λ value of 0.15 and the datasets of binocular advantage of error rate and speed were log transformed to achieve normality, thereby making them amenable to parametric statistics. Note, however, that Figures 3 to 6 containing the study results are all constructed on the raw untransformed data for visualization purposes. Two-factor repeated measures multiple analysis of variable (RM-MANOVA) was performed to investigate the between-subjects factor of cohort type (controls versus cases) and the within-subjects factor of viewing condition (binocular versus monocular) on the dependent variables of error rate and speed. A separate one-factor, between-subjects MANOVA was performed to compare the binocular advantage in error rate and speed between controls and cases. Similarly, a separate one-factor, within-subjects MANOVA was performed to compare the impact of optical correction modality (rigid contact lens versus spectacles) on stereo threshold, error rate, and speed.

### Comparison of Buzz-Wire Performance in Cases With Those of Uncorrected Myopes

The results from the main experiment revealed that the monocular and binocular buzz-wire task performance was worse in cases than in controls. An additional experiment was performed to determine whether this deterioration was unique to keratoconus or generic to any form of optical blur experienced by the individual – for instance, optical blur from uncorrected axial myopia, but with a regularly shaped cornea. This experiment tested the hypothesis that the error rate and speed in the buzz-wire task will be similar in cases and uncorrected myopic cohorts with comparable levels of visual acuity and stereo thresholds. Ten participants with −6.00 D to −13.00 D of uncorrected myopia (21–34 years) repeated the monocular and binocular versions of the buzz-wire task. All other details were identical to the main experiment.

## Results

Table 1 describes the demographic and clinical details of the study participants (see Appendix for individual cases). Ten of the 30 cases had bilateral keratoconus with similar severity in both eyes. The remaining cases were either bilateral keratoconus with different disease severities in the two eyes or those with a clinically manifest keratoconus in only one eye (see Appendix).

**Table 1.** Demographic and Clinical Details of Study Participants

	Cases (*n* = 30)	
	**Right Eye**	**Left Eye**
Age (years)	20 (17 to 34)	
Sex (M : F)	20 : 10	
D-index (unitless)	8.09 (2.13 to 27.13)	7.28 (0.53 to 22.05)
SER (D)	−3.50 (−12.00 to 0.00)	−3.50 (−24.00 to −0.38)
J_0_ (D)	0.00 (−2.59 to 2.82)	0.09 (−2.35 to 4.59)
J_45_ (D)	0.77 (−0.94 to 2.95)	−0.99 (−3.87 to 2.38)
BSCVA (logMAR)	0.30 (0.00 to 1.60)	0.30 (0.00 to 1.40)
Stereo threshold (arc sec)	547.13 (52.66 to 1906.00)	
	**Controls (*n* = 26)**	
	**Right Eye**	**Left Eye**

Age (years)	24 (17 to 29)	
Sex (M : F)	9 : 17	
D-index (unitless)	0.72 (−0.37 to 2.45)	0.76 (−1.16 to 2.61)
SER (D)	0.00 (−5.00 to 0.88)	0.00 (−5.00 to 0.88)
J_0_ (D)	0.00 (0.00 to 1.25)	0.00 (0.00 to 1.25)
J_45_ (D)	0.00 (0.00 to 0.00)	0.00 (0.00 to 0.32)
BSCVA (logMAR)	0.00 (0.00 to 0.00)	0.00 (0.00 to 0.00)
Stereo threshold (arc sec)	29.99 (3.18 to 77.70)	

BSCVA, best spectacle-corrected visual acuity.

The values indicate the median (minimum to maximum) for each parameter described in the study. The SER, J_0_, and J_45_ power vector terms represent the spherical equivalent of refraction and the regular and oblique astigmatic components of refraction, respectively.^34^

### Buzz-Wire Task Performance in Controls and Cases

Figure 3A shows scatter diagrams of the binocular and monocular error rate for controls and cases with their habitual spectacles. The error rate patterns in both cohorts resembled the schematic in Figure 2G. The orientation and the centroid locations of the bivariate contour ellipse for controls indicated a uniform shift in the data below the line of equality (Fig. 3A). In contrast, the bivariate contour ellipse for cases was steeper than 45 degrees, with its y-axis centroid remaining significantly lower than its x-axis centroid (Fig. 3A). Additionally, the rightward and upward shift in the x- and y-axes centroids, respectively, of cases, relative to controls, indicated an overall higher error rates in cases than in controls (Fig. 3A).

The bivariate contour ellipses for speed were oriented close to the 45-degree line of equality in controls and cases (Fig. 3B). For controls, the x-axis centroid of the ellipse was lower than the y-axis centroid, indicating a slowing down under monocular viewing condition (Fig. 3B), whereas in cases, the x- and y-axes centroids for cases were not different to each other (Fig. 3B), indicating that the cases did not slow down as much as the controls under monocular viewing. Additionally, the speed ellipse of cases was shifted rightward, relative to controls, suggesting that under monocular viewing, the former cohort performed the task faster than the latter cohort under monocular viewing conditions (Fig. 3B).

The Box-Cox transformed monocular error rates of controls and cases were higher than the binocular values (Table 2, Section 1a). The multivariate test in the two-factor RM-MANOVA revealed significant main effects of viewing condition and cohort and significant interaction between the two main effects on the combined dependent variables of error rate and speed (Table 2, Section 2a). These effects were retained in the univariate tests, with the effect size being stronger for the former than the latter outcome variable (Table 2, Section 2b). To further investigate the pattern of error rates obtained in cases, their monocular and binocular error rates were divided into two subgroups about the y-axis centroid, that is, participants with binocular error rates lower and higher than the y-axis centroid. The mean difference in the Box-Cox transformed monocular and binocular error rates was found to be significant only for the latter subgroup and not the former subgroup (Table 2, Section 1a).

**Table 2.** Results of the Main Statistical Analyses Conducted in this Study

**Section 1: *T*-Tests**								
	**1a. Paired *t*-tests**							
			**Error Rate**			**Speed**		
	**Cohort**	**Comparison Groups**	**Mean diff ± SEM**	**T value**	***p* Value**	**Mean Diff ± SEM**	**T value**	***p* Value**

Comparison of error rate and speed	Control	Bino vs. Mono	−1.00 ± 0.19	−5.23	**<0.001**	0.26 ± 0.06	4.26	**<0.001**
among controls and cases	KC	Bino vs. Mono	−0.44 ± 0.09	−4.92	**<0.001**	0.03 ± 0.05	0.5	0.62
	KC below threshold	Bino vs. Mono	0.71 ± 0.10	−7.11	**<0.001**	—		
	KC above threshold	Bino vs. Mono	0.08 ± 0.04	−1.93	0.07			
**Section 2: Two-factor RM-MANOVA Analysis**								
	**2a. Multivariate tests**							
	**Factors**			**F**		***p* value**		**Partial ƞ^2^**
	
Effect of viewing condition (Bino vs.	Viewing condition			41.8		**<0.001**		0.61
Mono) and cohort type (control vs.	Cohort type			9.33		**<0.001**		0.26
cases) on error rate and speed	Viewing condition × cohort type			11.72		**<0.001**		0.31
	**2b. Univariate tests**							
			**Error rate**			**Speed**		
	**Factors**	**Group**	**Mean ± SEM**	***p* value**	**Partial ƞ^2^**	**Mean ± SEM**	***p* value**	**Partial ƞ^2^**
	
	Viewing condition	Binocular	−1.73 ± 0.11	**<0.001**	0.48	0.50 ± 0.06	**<0.001**	0.24
		Monocular	−1.03 ± 0.03			0.36 ± 0.06		
	Cohort type	Controls	−1.66 ± 0.10	**<0.001**	0.26	0.31 ± 0.08	**0.03**	0.08
		KC	−1.10 ± 0.10			0.55 ± 0.08		
	Viewing condition × cohort type	—	—	**0.004**	0.15	—	**0.004**	0.14
**Section 3: One-factor RM-MANOVA Analysis**								
	**3a. Multivariate tests**							
				**F**		***p* value**		**Partial ƞ^2^**
				
Binocular advantage in error rate and speed among control and cases	Cohort type			13.06		**<0.001**		0.33
	**3b. Univariate tests**							
			**Error rate**			**Speed**		
			**Mean ± SEM**	***p* value**	**Partial ƞ^2^**	**Mean ± SEM**	***p* value**	**Partial ƞ^2^**

	Cohort type	Controls	0.53 ± 0.06	**<0.001**	0.21	0.14 ± 0.03	**0.003**	0.13
		KC	0.22 ± 0.06			0.02 ± 0.03		
**Section 4: One-factor MANOVA Analysis**								
	**4a. Multivariate tests**							
				**F**		***p* value**		**Partial ƞ^2^**
				
Effect of spectacle and contact lenses on stereo, error rate and speed	Correction modality			163.89		**<0.001**		0.97
	**4b. Univariate tests**							
			**Stereo threshold**		**Error rate**		**Speed**	
			**Mean ± SEM**	***p* value**	**Mean ± SEM**	***p* value**	**Mean ± SEM**	***p* value**

	Correction modality	Spectacle	2.71 ± 0.12	**0.001**	−1.23 ± 0.12	**<0.001**	0.69 ± 0.12	**0.001**
		Contact lens	2.34 ± 0.12		0.76 ± 0.02		1.10 ± 0.01	
**Section 5: One-factor RM-MANOVA Analysis**								
	**5a. Multivariate tests**							
				**F**		***p* value**		**Partial ƞ^2^**
				
Effect of viewing on error rate and speed among uncorrected myopes	Viewing condition			23.06		**0.001**		0.72
	**5b. Univariate tests**							
			**Error rate**			**Speed**		
			**Mean ± SEM**	***p* value**	**Partial ƞ^2^**	**Mean ± SEM**	***p* value**	**Partial ƞ^2^**

	Viewing condition	Binocular	−1.56 ± 0.12	**0.001**	0.72	0.16 ± 0.15	**0.003**	0.64
		Monocular	−0.99 ± 0.05			0.08 ± 0.19		
**Section 6: Mann-Whitney test**								
			**Error rate**			**Speed**		
	**Factor**	**Comparison groups**	**Median (IQR)**	**Z**	***p* value**	**Median (IQR)**	**Z**	***p* value**

Bino advantage myopes versus cases	Cohort type	Myopes	1.98 (1.37 – 2.32)	−2.26	**0.02**	1.27 (1.09 – 1.53)	−2.72	**0.005**
with comparable stereo loss		Cases	1.16 (0.99 – 1.81)			1.03 (0.87 – 1.12)		

Bino, binocular; KC, keratoconus; Mono, monocular.

Section 1 shows the results of *t*-tests comparing the binocular versus monocular performances of controls and cases. Sections 2a and b show the results of the multivariate and univariate two-factor RM-MANOVA comparing the binocular and monocular task performances of controls and cases, respectively. Sections 3a and b show the results of the multivariate and univariate one-factor RM-MANOVA comparing the binocular advantages for the two outcome variables in controls and cases. Sections 4a and b show the results of the multivariate and univariate one-factor MANOVA comparing the impacts of correction modality on the stereoacuity and error rate of cases. Sections 5a and b show the results of the multivariate and univariate one-factor MANOVA reporting the impacts of viewing condition on the error rate and speed in uncorrected myopes. Section 6 shows the results of the Mann-Whitney test comparing the binocular advantages in error rates and speed among uncorrected myopes and cases with comparable stereo loss. The mean ± standard error of the mean (SEM) shown here under sections 1, 2, 4 and 5 are the Box-Cox transformed values, as described in the Methods section. The mean values shown here may be retransformed to its raw form by using the formula:x raw =λx trans +1λ, where  x_raw_ is the mean of the raw data, x_trans_ is the mean of the transformed data, and λ is the Box-Cox transformation exponent used in this study (λ = 0.15). The mean ± SEM under section 3 are the log transformed values as mentioned in the method section. Relationships with *p* < 0.05 (uncorrected for multiple comparisons) appear in bold.

The one-factor MANOVA performed on the log-transformed binocular advantage scores showed a significant difference between controls and cases for the combined dependent variables (Figs. 3C, 3D; Table 2, Section 3a). The univariate tests showed that the binocular advantages in error rate and speed were higher in controls than in cases, with the effect size being higher for error rate than speed (Table 2, Section 3b). These trends were expected from the binocular and monocular data of these outcome variables reported in Table 2, Sections 1 and 2.

### Relationship Between Stereo Threshold and Binocular Advantage in Error Rate

Unlike controls, the addition of binocularity had a differential impact on error rates of cases (Fig. 3A). To determine if this pattern was related to the participants’ stereo thresholds, the binocular advantages in error rates of cases were plotted against their stereo thresholds (Fig. 4). The same relationship for controls is also shown in this figure for comparison. All controls had stereo thresholds lower than the buzz-wire task's diastereopsis threshold (vertical line in Fig. 4), making the task a suprathreshold activity. Whereas all the controls showed a distinct binocular advantage in error rate, this advantage was poorly correlated with stereo threshold (Pearson's *r* = −0.25, *p* = 0.22). Only 10 cases had stereo thresholds lower than the diastereopsis threshold, all of whom also showed a binocular advantage in the error rate (Fig. 4). Among the remaining 20 cases with stereo thresholds poorer than the diastereopsis disparity threshold, 10 exhibited near unity binocular advantage, 3 had binocular advantage comparable to that of controls, and the binocular advantage of the rest was somewhere in between (Fig. 4). Overall, like controls, there was a non-significant correlation between binocular advantage in error rate and stereo threshold in the cases (Pearson's *r* = −0.32, *p* = 0.08).

Among cases, binocular advantage in error rate poorly correlated with the two eyes’ maximum D-index, the difference between the two eyes’ D-indices, and the maximum, mean, and the difference between the two eyes’ best-corrected visual acuity (*r* ≤ −0.32, *p* ≥ 0.08, for all).

### Impact of Rigid Contact Lenses on the Error Rates of the Cases

With rigid contact lens wear, the stereo threshold and error rate of cases were below the 1:1 line, indicating an improvement in these variables relative to spectacles (Figs. 5A, 5B; Table 2, Section 4b). The one-factor MANOVA showed a statistically significant impact of the correction modality for the combined dependent variables (Table 2, Section 4a). The univariate tests confirmed this effect for both stereo threshold and error rate, with the effect size being larger for the latter than the former variable (Table 2, Section 4b). However, the proportional improvements in stereo threshold and error rate, obtained by dividing the value obtained with spectacles by the value obtained with contact lenses, proved to be uncorrelated (Pearson's *r* = 0.02, *p* = 0.94; Fig. 5C).

### Buzz-Wire Task Performance of Uncorrected Myopes

Visual acuities among the uncorrected myopes (0.91 ± 0.07 logMAR) were significantly poorer than among those cases that were above the diastereopsis threshold (0.50 ± 0.07 logMAR; *t* = 4.01, *p* = 0.001). Stereo thresholds, on the other hand, were comparable between the two cohorts (uncorrected myopes = 3.28 ± 0.20 log arc sec and cases = 3.20 ± 0.06 log arc sec, *t* = 0.43, *p* = 0.67 see the blue versus red bubbles in Fig. 4).

Scatter diagrams of error rate and speed for the participants with uncorrected myopia have been fit with bivariate contour ellipses and superimposed on the corresponding ellipses for controls and cases in Figure 6. The bivariate contour ellipse for error rates in the uncorrected myopes was oriented at 57.9 degrees, with its x-axis centroid remaining higher than its y-centroid (Fig. 6A). The one-factor RM-MANOVA analysis showed a significant impact of viewing condition on the combined dependent variable (Table 2, Section 5a) and the univariate tests confirmed a significant impact of viewing condition for both error rates and speed (Table 2, Section 5b). The log-transformed binocular advantage in error rate (mean ± SEM = 0.22 ± 0.04) was well correlated with logMAR visual acuity (Pearson's *r* = −0.73, *p* = 0.02, data not shown) but poorly correlated with stereo threshold (Pearson's *r* = −0.09, *p* = 0.80; Fig. 4). The binocular advantages in error rate and speed were also significantly higher among uncorrected myopes than among cases with comparable levels of stereo threshold (Fig. 4; Table 2, Section 6).

## Discussion

### Summary of Results

1. The controls made fewer errors when viewing the buzz wire binocularly (Table 2; Fig. 3A). However, only those cases with relatively low monocular error rates showed a similar advantage from binocular viewing (Fig. 3A). Cases with high monocular error rates also had higher error rates when viewing the buzz wire binocularly (Table 2; Fig. 3A).
2. An improvement in the retinal image quality of cases with rigid contact lens wear reduced the binocular error rates in the buzz-wire task, vis-à-vis, spectacles (Fig. 5B).
3. Two observations indicate that psychophysical estimates of stereo thresholds may not be a good predictor of error rates in visuomotor activities like the buzz-wire task. First, stereo threshold proved to be poorly correlated with the binocular advantage in the error rate among the participants within each cohort. Second, stereo threshold proved to be poorly correlated with the reduction in error rate enjoyed by the cases, when they switched from their best-corrected spectacles to contact lenses (Fig. 5C).
4. Controls, uncorrected myopes, and cases executed the buzz-wire task faster under binocular than monocular conditions (Figs. 3B, 6B). However, the magnitude of speed reduction from binocular to monocular viewing was smaller in cases than in the controls and uncorrected myopes (Figs. 3B, 6B; Table 2).

These results compare well with previous findings of deficient visuomotor task performance in other forms of ophthalmic disease, such as amblyopia and strabismus,^7^^,^^8^ and indicate that functional depth vision may be severely compromised with degraded binocularity, irrespective of the cause of this dysfunction. Finally, these results also align well with those of Knill, who showed that visuomotor tasks like hand reaching are heavily weighted toward the binocular retinal disparity cue, with little influence of monocular cues on task performance.^3^

### Stereo Threshold as Poor Predictor of Visuomotor Task Performance

There are at least two reasons why the psychophysical stereo threshold may correlate poorly with error rate in the buzz-wire task. First, the executive requirements of the random-dot stereogram task and the buzz-wire task may be quite different.^35^ The former is a hyperacuity task, requiring good quality correspondence matching of the monocular images for fusion, computation of retinal disparity from the fused percept, and an inference about the geometric shape of the 3D object in an otherwise two-dimensional field of random dots.^36^ The buzz-wire task, on the other hand, relies on accurate and continuous judgment of the diastereopsis of a physical 3D structure that guides hand movements to avoid contact between the loop and the wire in the task.^5^ These two measures may respond very differently to the degraded retinal image quality experienced in the present study. Random-dot stereo targets may be more vulnerable to the contrast loss and phase distortions in the blurred retinal image,^15^^,^^37^ reaching stereo-blindness levels when thresholds exceed 1300 arc sec,^38^ whereas useful information regarding diastereopsis may still be available in the buzz-wire task for comparable levels of blur. Evidence for this possibility arises from the uncorrected myopes continuing to show a binocular advantage in the buzz-wire task, even while they were all nearly stereo-blind (Figs. 4, 6A). This binocular advantage may be derived from non-stereoscopic cues that may aid the identification of the gap between the loop and the wire in this task, unlike random-dot stereograms that are entirely reliant on the retinal disparity cue for stereo processing. However, the prominent monocular cue of motion parallax derived from head movements may not be useful for depth judgments in the buzz-wire task, as reported recently by Devi et al.^5^ The complexity of integrating retinal image motion arising from head velocity with the velocity of object motion arising from passing the loop through the buzz wire may make this cue less beneficial to the present task performance.^5^

The second reason is that the stereoscopic information in a random-dot target is to be inferred from a two-dimensional field of random dots might make this task more unnatural and, thus, more vulnerable to retinal image quality degradation. On the contrary, the buzz-wire task is similar to routine depth-related activities of daily living wherein the stereoscopic information is derived from objects that are physically separated in space. Perhaps a top-down knowledge of the buzz-wire configuration, and/or the depth information derived from convergence eye movements while tracking the depth convoluted buzz-wire makes this task less vulnerable to retinal image quality degradation.^39^ After all, our ability to generate accurate vergence eye movements remains largely unaffected in the presence of either iso-ametropic or anisometropic retinal image blur.^40^ Future studies could employ depth judgments between physically separated objects to determine the relationship between stereo thresholds and errors in the buzz-wire task.

### Retinal Image Quality and its Impact on Visuomotor Task Performance

**Figure 7.**
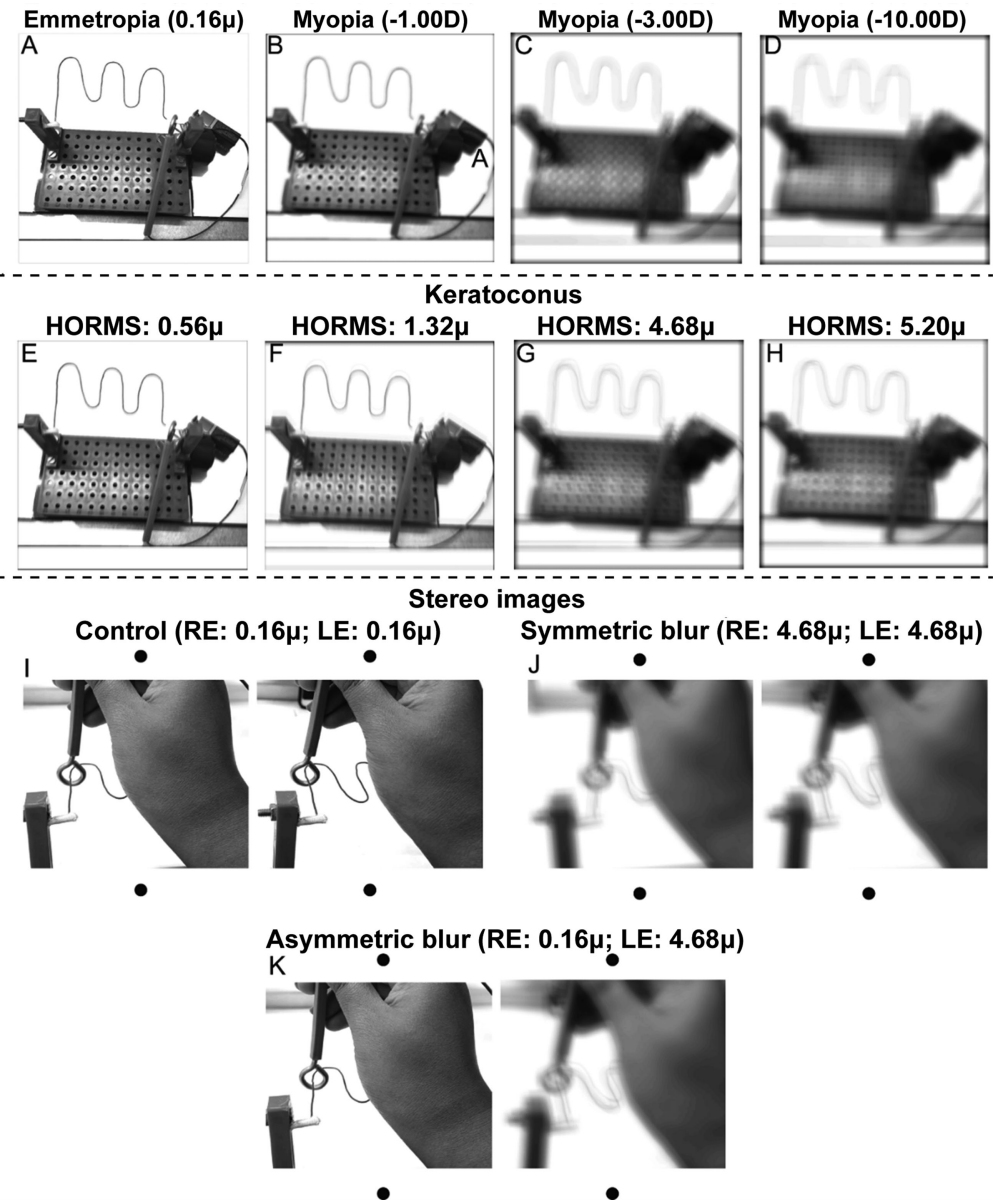
(**A–H**) Point-of-view optical simulations of the buzz-wire apparatus with clear vision (**A**), blurred vision from uncorrected myopia (**B–D**) and blurred vision from cases, whose severity is indicated on top of each panel by the root-mean-squared values of the higher-order aberrations (HORMS) (**E–H**). (**I–K**) Show cross-fusible zoomed-in stereoscopic image pairs of the buzz-wire apparatus illustrating the location of the loop relative to the wire when vision is clear in both eyes (**I**) and when vision is bilaterally (**J**) or unilaterally (**K**) blurred from keratoconus. The wavefront aberration values used to blur the right eye (RE) and left eye (LE) of the stereogram are indicated in each figure panel.

The nature of blur experienced by the participants and its bilateral (a)symmetry could have a determining impact on the buzz-wire task investigated in this study. Deeper insights into this issue may be obtained through simulation of how the buzz-wire apparatus may appear from the blur in cases, uncorrected myopia, and in controls (Fig. 7). All the following simulations were performed for 555 nm light and 5 mm pupil diameter, using standard Fourier optics techniques.^41^ The point spread function (PSF) of the eye with clear vision was generated using only population average higher-order Zernike wavefront aberrations obtained from Cheng et al.^42^ (Fig. 7A). The PSFs of uncorrected myopes were generated by adding 1 D, 3 D, and 10 D worth of defocus to the population average higher-order Zernike aberrations (Figs. 7B–D, respectively). Case PSFs are obtained from higher-order Zernike aberrations, corresponding to early, mild, moderate, and severe keratoconus already available in the laboratory (Figs. 7E–H).^12^ Lower-order aberrations are assumed to be fully corrected in keratoconus, whereas in reality, some may remain owing to variability in estimating the subjective refraction endpoint.^43^^,^^44^

The uncorrected myopes in the present study were all iso-ametropic, resulting in similar magnitudes of radially symmetric blur in the two eyes. This radial symmetric blur is characterized largely by contrast demodulations while retaining the spatial relationship between the loop and the wire (Figs. 7B–D). The bilateral symmetry of blur continues to support the fusion of the monocular percept (Fig. 7J). Both features may help retain the diastereopsis information under binocular viewing in uncorrected myopia (Fig. 7J). In contrast, the keratoconus cases experience radially anisotropic blur that may also be bilaterally dissimilar owing to differences in disease severity between the eyes (Appendix Table A1). The radially asymmetric blur introduces significant phase distortions that disrupt the spatial relationship between the loop and the wire under monocular viewing (manifesting as “ghosting” or “doubling” of the wire in Figs. 7E–H).^15^ Binocularly, the phase distortions may disrupt the correspondence matching between the monocular precepts^14^ and the bilaterally asymmetric blur may induce interocular suppression of the more blurred percept,^17^ both of which may lead to poor quality diastereopsis (Fig. 7K). These effects may explain the absence of binocular advantage in the buzz-wire task for the cases, even while it was retained in the uncorrected myopes (Fig. 6). The improved buzz-wire task performance in rigid contact lens cases relative to spectacle cases may have been by reducing the contrast demodulation and phase disruption in the monocular retinal images and by improving the symmetry in the retinal image quality of the two eyes.^15^^,^^17^^,^^37^ A future study could compare the buzz-wire task performance in uncorrected anisometropia and bilaterally asymmetric keratoconus to gain deeper insights into this issue. The improved error rates in the buzz-wire task of cases with rigid contact lenses could also be a learning effect, as the buzz-wire task was first performed with spectacles and then with contact lenses. However, Devi et al.^5^ investigated this possibility and found no evidence of a learning effect over the three trials. Nonetheless, future studies may systematically investigate the impact of any learning effect on the buzz-wire task performance.

### Clinical Implications

The present results suggest that keratoconus may increase the difficulty in executing activities of daily living that involve 3D depth judgments (e.g. driving, navigating obstacles, and climbing stairs; Fig. 3). These factors, combined with their suboptimal spatial vision,^11^ may contribute toward an overall deterioration in their quality of life and general well-being.^45^ Rigid contact lenses that improve retinal image quality may be one way to minimize this deterioration (Fig. 5B). Interestingly, neither the disease severity nor the routinely evaluated clinical measures of visual acuity or stereoacuity were good predictors of such visuomotor activity limitations (Fig. 4). This observation, on one hand, reveals the limitation of the clinical measures in reflecting the real-world visual experience of the patient, and, on the other hand, underlines the need for expanding the visual assessment battery to include measures that emulate the complexities of daily tasks.

The lack of a prominent speed reduction in the buzz-wire task in keratoconus is contrary to the expectation of how this parameter may decline in the presence of uncertain sensory inputs (arising from blurred vision and poor stereopsis, in this case^11^^,^^18^). This may be so for two reasons. First, the binocular and monocular viewing experience in keratoconus in such tasks may be similar, given their habitually suboptimal vision. Thus, there may be no overt reason to decrease the speed under monocular viewing, relative to binocular viewing. Second, keratoconics may harbor false beliefs that they can see well in depth despite their degraded binocularity. This may reflect a general personality trait of keratoconics and the difficulties they may experience coping with vision loss.^46^^,^^47^ These hypotheses need further investigation.

## App Appendix

**Table A1.** Demographic Details of the 30 Keratoconic Participants Along With Their Corneal Topographic Outcomes (Maximum Keratometry and D–Index) and Visual Functions (logMAR Visual Acuity and Stereo Thresholds) With Spectacles and Contact Lens

			Maximum Keratometry *(D)*		D–Index *(Unitless)*		Visual Acuity With Spectacles *(logMAR)*			Visual Acuity With Contact Lens *(logMAR)*		
Sub No.	Age, Y	Sex	RE	LE	RE	LE	RE	LE	Stereo Threshold With Spectacles (Arc Sec)	RE	LE	Stereo Threshold With Contact Lens (Arc Sec)
1	19	M	45.5	70.8	2.96	16.77	0.00	0.60	841.97	^*^	0.10	820.65
2	18	M	44.6	79.3	2.13	22.05	0.00	1.10	3372.25	^*^	0.40	1032.86
3	19	M	55.5	44.1	8.06	0.53	1.40	0.00	800.18	0.00	0.00	62.03
4	19	M	58.5	51.4	7.97	5.51	0.30	0.18	462.70	0.30	0.00	142.78
5	22	M	56.7	48.8	7.94	4.29	0.40	0.00	222.54	0.18	0.00	33.94
6	22	F	67.6	50.7	14.2	6.44	0.70	0.00	1159.30	0.00	^*^	233.76
7	20	F	44.9	43.0	9.41	4.12	0.18	0.00	63.31	0.10	0.00	56.88
8	25	M	56.6	57.0	7.19	7.89	0.00	0.30	281.38	^*^	0.00	127.48
9	26	F	48.6	55	3.87	6.89	0.00	0.10	60.62	0.00	0.00	192.58
10	17	M	60.1	52.6	11.09	5.89	0.30	0.18	278.80	0.00	0.00	401.12
11	26	F	61.3	57.1	14.96	16.32	0.30	0.18	584.58	0.00	0.00	145.83
12	32	M	48.5	64.6	7.27	12.8	0.18	0.48	251.90	^*^	0.00	138.43
13	18	M	62.4	75.5	8.38	16.95	0.18	0.30	1390.05	0.00	0.00	1115.27
14	21	F	53.6	41.4	10.12	3.36	0.70	0.10	1997.88	0.00	^*^	601.26
15	18	M	56.8	49.8	11.96	6.83	0.40	0.18	855.42	0.00	0.00	169.57
16	24	M	56.1	61.9	9.03	8.7	0.00	0.40	717.63	0.10	0.00	296.03
17	24	M	49.7	50	6.99	6.52	0.18	0.10	200.85	0.00	0.10	134.62
18	25	F	54.4	52.7	4.85	4.63	0.18	0.18	666.34	NA	NA	NA
19	17	M	48.5	55.6	3.38	7.67	0.30	0.30	1855.71	NA	NA	NA
20	34	F	53.2	53.1	4.99	3.58	0.30	0.30	1773.45	NA	NA	NA
21	22	M	63.6	60.2	11.29	10.2	0.18	0.10	146.20	NA	NA	NA
22	28	M	52.9	42.8	5.31	1.35	0.48	0.48	250.76	NA	NA	NA
23	19	M	63.3	55.2	14.44	8.33	0.48	0.18	601.53	NA	NA	NA
24	22	M	44	46	2.27	5.17	0.30	0.18	139.60	NA	NA	NA
25	19	F	65.9	44.6	NA	4	1.10	0.00	4596.21	NA	NA	NA
26	24	F	71.3	50.8	24.6	16.3	0.90	0.18	1118.56	NA	NA	NA
27	20	M	62.4	53.5	11.67	11.24	0.60	0.48	1720.88	NA	NA	NA
28	18	M	62.4	52.5	27.13	10.17	1.60	0.80	1269.90	NA	NA	NA
29	28	F	46.9	54.4	8.09	10.71	0.30	0.40	1927.61	NA	NA	NA
30	17	M	48.9	52.3	9.89	12.49	0.70	1.10	1048.99	NA	NA	NA

CL, contact lens; LE, left eye; max K, maximum keratometry reading; NA, not applicable (these participants were not tested with contact lens correction). Participants 1 – 17 performed the task with both spectacles and contact lenses; RE, right eye; SP, spectacle; stereo, stereo threshold.

* The asterisk symbol indicates participants wore contact lens only in one eye, for which the visual acuity is reported. The fellow eye's refractive error was corrected with spectacles, if any. The fellow eye's acuity thus equaled what is reported in columns 8 and 9 of this table. Participants 18 to 30 performed the task with only their spectacles.

## References

[1] Read JC, Begum SF, McDonald A, Trowbridge J. The binocular advantage in visuomotor tasks involving tools. *Iperception*. 2013; 4: 101–110. 23755355 10.1068/i0565 PMC3677330

[2] Fielder AR, Moseley MJ. Does stereopsis matter in humans? *Eye (Lond)*. 1996; 10: 233–238. 8776453 10.1038/eye.1996.51

[3] Knill DC. Reaching for visual cues to depth: the brain combines depth cues differently for motor control and perception. *J Vis*. 2005; 5: 103–115. 15831071 10.1167/5.2.2

[4] Wheatstone C. On some remarkable and hitherto unobserved phenomena of binocular vision. *Phil Trans Roy Soc, London*. 1838; 128: 371–394. 14000225

[5] Devi P, Solomon JA, Tyler CW, Dave TV, Kaliki S, Bharadwaj SR. Comparison of depth-related visuomotor task performance in uniocular individuals and in binocular controls with and without temporary monocular occlusion. *Invest Ophthalmol Vis Sci*. 2024; 65: 32. 10.1167/iovs.65.8.32 PMC11262539 39028979

[6] Piano ME, O'Connor AR. The effect of degrading binocular single vision on fine visuomotor skill task performance. *Invest Ophthalmol Vis Sci*. 2013; 54: 8204–8213. 24222309 10.1167/iovs.12-10934

[7] O'Connor AR, Birch EE, Anderson S, Draper H. Relationship between binocular vision, visual acuity, and fine motor skills. *Optom Vis Sci*. 2010; 87: 942–947. 21057348 10.1097/OPX.0b013e3181fd132e

[8] O'Connor AR, Birch EE, Anderson S, Draper H, Group F. The functional significance of stereopsis. *Invest Ophthalmol Vis Sci*. 2010; 51: 2019–2023. 19933184 10.1167/iovs.09-4434

[9] Verghese P, Tyson TL, Ghahghaei S, Fletcher DC. Depth perception and grasp in central field loss. *Invest Ophthalmol Vis Sci*. 2016; 57: 1476–1487. 27031841 10.1167/iovs.15-18336 PMC4819556

[10] Santodomingo-Rubido J, Carracedo G, Suzaki A, Villa-Collar C, Vincent SJ, Wolffsohn JS. Keratoconus: an updated review. *Cont Lens Anterior Eye*. 2022; 45: 101559. 34991971 10.1016/j.clae.2021.101559

[11] Kumar P, Campbell P, Vaddavalli PK, Hull CC, Bharadwaj SR. Structure-function relationship in keratoconus: Spatial and depth Vision. *Transl Vis Sci Technol*. 2023; 12: 21. 10.1167/tvst.12.12.21 PMC10756247 38149965

[12] Nilagiri VK, Metlapally S, Schor CM, Bharadwaj SR. A computational analysis of retinal image quality in eyes with keratoconus. *Sci Rep*. 2020; 10: 1321. 31992755 10.1038/s41598-020-57993-w PMC6987247

[13] Pantanelli S, MacRae S, Jeong TM, Yoon G. Characterizing the wave aberration in eyes with keratoconus or penetrating keratoplasty using a high-dynamic range wavefront sensor. *Ophthalmology*. 2007; 114: 2013–2021. 17553566 10.1016/j.ophtha.2007.01.008

[14] Metlapally S, Bharadwaj SR, Roorda A, Nilagiri VK, Yu TT, Schor CM. Binocular cross-correlation analyses of the effects of high-order aberrations on the stereoacuity of eyes with keratoconus. *J Vis*. 2019; 19: 12. 10.1167/19.6.12 PMC6559754 31185094

[15] Marella BL, Conway ML, Vaddavalli PK, Suttle CM, Bharadwaj SR. Optical phase nullification partially restores visual and stereo acuity lost to simulated blur from higher-order wavefront aberrations of keratoconic eyes. *Vision Res*. 2024; 224: 108486. 39298859 10.1016/j.visres.2024.108486

[16] Ferdi AC, Nguyen V, Gore DM, Allan BD, Rozema JJ, Watson SL. Keratoconus natural progression: a systematic review and meta-analysis of 11 529 eyes. *Ophthalmology*. 2019; 126: 935–945. 30858022 10.1016/j.ophtha.2019.02.029

[17] Marella BL, Conway ML, Suttle C, Bharadwaj SR. Contrast rivalry paradigm reveals suppression of monocular input in keratoconus. *Invest Ophthalmol Vis Sci*. 2021; 62: 15. 10.1167/iovs.62.2.12 PMC7884294 33570601

[18] Nilagiri VK, Metlapally S, Kalaiselvan P, Schor CM, Bharadwaj SR. LogMAR and stereoacuity in keratoconus corrected with spectacles and rigid gas-permeable contact lenses. *Optom Vis Sci*. 2018; 95: 391–398. 29554011 10.1097/OPX.0000000000001205 PMC5968352

[19] Dandapani SA, Padmanabhan P, Hussaindeen JR. Spectrum of binocular vision anomalies in keratoconus subjects. *Optom Vis Sci*. 2020; 97: 424–428. 32511164 10.1097/OPX.0000000000001517

[20] Deshmukh R, Ong ZZ, Rampat R, et al. Management of keratoconus: an updated review. *Front Med (Lausanne)*. 2023; 10: 1212314. 37409272 10.3389/fmed.2023.1212314 PMC10318194

[21] Scheiman M, Wick B. *Clinical management of binocular vision: heterophoric, accommodative, and eye movement disorders*. New York, NY: Lippincott Williams & Wilkins; 2008.

[22] Faul F, Erdfelder E, Lang A-G, Buchner A. G* Power 3: a flexible statistical power analysis program for the social, behavioral, and biomedical sciences. *Behav Res Methods.* 2007; 39: 175–191. 10.3758/bf03193146 17695343

[23] Gonzalez E, Steinbach M, Ono H, Wolf M. Depth perception in children enucleated at an early age. *Clin Vis Sci*. 1989; 4: 173–177.

[24] Cohen J. *Statistical power analysis for the behavioral sciences*. 2 ed: Hillsdale, NJ: Lawrence Erlbaum Associates, Publishers; 1988.

[25] Downie LE, Lindsay RG. Contact lens management of keratoconus. *Clin Exp Optom*. 2015; 98: 299–311. 26104589 10.1111/cxo.12300

[26] Hashemi H, Beiranvand A, Yekta A, Maleki A, Yazdani N, Khabazkhoob M. Pentacam top indices for diagnosing subclinical and definite keratoconus. *J Curr Ophthalmol*. 2016; 28: 21–26. 27239598 10.1016/j.joco.2016.01.009 PMC4881219

[27] Muftuoglu O, Ayar O, Hurmeric V, Orucoglu F, Kilic I. Comparison of multimetric D index with keratometric, pachymetric, and posterior elevation parameters in diagnosing subclinical keratoconus in fellow eyes of asymmetric keratoconus patients. *J Cataract Refract Surg*. 2015; 41: 557–565. 25708211 10.1016/j.jcrs.2014.05.052

[28] Granados-Delgado P, Casares-Lopez M, Martino F, Anera RG, Castro-Torres JJ. The role of visual performance in fine motor skills. *Life (Basel)*. 2024; 14: 1354. 39598153 10.3390/life14111354 PMC11595507

[29] Hou SW, Zhang Y, Christian L, Niechwiej-Szwedo E, Giaschi D. Evaluating visuomotor coordination in children with amblyopia. *Dev Psychobiol*. 2022; 64: e22270. 35452551 10.1002/dev.22270

[30] Brainard DH . The Psychophysics Toolbox. *Spat Vis*. 1997; 10: 433–436. 9176952

[31] Watson AB. Probability summation over time. *Vision Res*. 1979; 19: 515–522. 483579 10.1016/0042-6989(79)90136-6

[32] Jinabhai A, O'Donnell C, Radhakrishnan H. Changes in refraction, ocular aberrations, and corneal structure after suspending rigid gas-permeable contact lens wear in keratoconus. *Cornea*. 2012; 31: 500–508. 22314817 10.1097/ICO.0b013e31820f777b

[33] Gillay Z. Plot ellipse on scattered 2D data. https://inmathworkscom/matlabcentral/fileexchange/116610-plot-ellipse-on-scattered-2d-data Accessed on September 10, 2024, and August 25, 2022.

[34] Thibos LN, Wheeler W, Horner D. Power vectors: an application of Fourier analysis to the description and statistical analysis of refractive error. *Optom Vis Sci*. 1997; 74: 367–375. 9255814 10.1097/00006324-199706000-00019

[35] Stevenson SB, Cormack LK, Schor CM. Hyperacuity, super resolution and gap resolution in human stereopsis. *Vision Res*. 1989; 29: 1597–1605. 2635483 10.1016/0042-6989(89)90141-7

[36] Julesz B. Binocular depth perception of computer-generated patterns. *Bell System Tech J*. 1960; 39: 1125–1162.

[37] Marella BL, Vaddavalli PK, Reddy JC, Conway ML, Suttle CM, Bharadwaj SR. Interocular contrast balancing partially improves stereoacuity in keratoconus. *Optom Vis Sci*. 2023; 100: 239–247. 36856557 10.1097/OPX.0000000000002001

[38] Chopin A, Bavelier D, Levi DM. The prevalence and diagnosis of 'stereoblindness' in adults less than 60 years of age: a best evidence synthesis. *Ophthalmic Physiol Opt*. 2019; 39: 66–85. 30776852 10.1111/opo.12607

[39] Rogers B. When is a disparity not a disparity? Toward an old theory of three-dimensional vision. *Iperception*. 2023; 14: 20416695231202726. 38812612 10.1177/20416695231202726 PMC11134170

[40] Bharadwaj SR, Candy TR. Accommodative and vergence responses to conflicting blur and disparity stimuli during development. *J Vis*. 2009; 9:4 1–18. 20053067 10.1167/9.11.4 PMC3971876

[41] Jaskulski M, Thibos L, Bradley A, Kollbaum P. IRIS – Indiana Retinal Image Simulator. 2019. Available at: https://blogs.iu.edu/corl/iris.

[42] Cheng H, Barnett JK, Vilupuru AS, et al. A population study on changes in wave aberrations with accommodation. *J Vis*. 2004; 4: 272–280. 15134474 10.1167/4.4.3

[43] Davis LJ, Schechtman KB, Begley CG, Shin JA, Zadnik K. Repeatability of refraction and corrected visual acuity in keratoconus. The CLEK Study Group. Collaborative Longitudinal Evaluation of Keratoconus. *Optom Vis Sci*. 1998; 75: 887–896. 9875994 10.1097/00006324-199812000-00011

[44] Raasch TW, Schechtman KB, Davis LJ, Zadnik K, CLEK Study Group. Collaborative Longitudinal Evaluation of Keratoconus Study. Repeatability of subjective refraction in myopic and keratoconic subjects: results of vector analysis. *Ophthalmic Physiol Opt*. 2001; 21: 376–383. 11563425 10.1046/j.1475-1313.2001.00596.x

[45] Gothwal VK, Gujar R, Sharma S, Begum N, Pesudovs K. Factors affecting quality of life in keratoconus. *Ophthalmic Physiol Opt*. 2022; 42: 986–997. 35638140 10.1111/opo.13010

[46] Aiello F, Gallo Afflitto G, Ceccarelli F, et al. Keratoconus and personality traits: a case-control study. *Cornea*. 2024; 43: 237–244. 37018764 10.1097/ICO.0000000000003284

[47] Mannis MJ, Ling JJ, Kyrillos R, Barnett M. Keratoconus and personality - a review. *Cornea*. 2018; 37: 400–404. 29215397 10.1097/ICO.0000000000001479

